# Urinary Extracellular Vesicles as Source of Biomarkers in Kidney Diseases

**DOI:** 10.3389/fimmu.2015.00006

**Published:** 2015-01-30

**Authors:** Ana Gámez-Valero, Sara Inés Lozano-Ramos, Ioana Bancu, Ricardo Lauzurica-Valdemoros, Francesc E. Borràs

**Affiliations:** ^1^IVECAT-Group, Institut d’Investigació Germans Trias i Pujol (IGTP), Badalona, Spain; ^2^Nephrology Service, Germans Trias i Pujol University Hospital, Badalona, Spain

**Keywords:** urinary extracellular vesicles, kidney disease, biomarker, therapy, isolation

## Abstract

Most cells physiologically release vesicles as way of intercellular communication. The so-called Extracellular Vesicles (EVs) include exosomes, ectosomes, and apoptotic bodies, which basically differ in their composition and subcellular origin. Specifically, EVs found in urine reflect the state of the urinary system, from podocytes to renal-tubular cells, thus making them an excellent source of samples for the study of kidney physiology and pathology. Several groups have focused on defining biomarkers of kidney-related disorders, from graft rejection to metabolic syndromes. So far, the lack of a standard protocol for EVs isolation precludes the possibility of a proper comparison among the different biomarkers proposed in the literature, stressing the need for validation of these biomarkers not only in larger cohorts of patients but also considering the different methods for EVs isolation. In this review, we aim to gather the current knowledge about EVs-related biomarkers in kidney diseases, with a special emphasis in the methods used to date for EVs enrichment, and discussing the need for more specific protocols of EV isolation in clinical practice.

## Introduction

Extracellular Vesicles (EVs) have awakened the interest of the scientific community due to their different roles in intercellular communication, pathogenesis, drug, and gene vector delivery, and as possible reservoirs of biomarkers (1–3). These vesicles can be released from different kind of cells, from platelets to cells of the immune system and neurons, among others (4), and can be found in several body fluids, including plasma, urine, and saliva (5, 6).

The term EVs includes different types of vesicles, mainly exosomes (EXs), ectosomes, and apoptotic bodies. These distinct types of vesicles differ in several aspects (7, 8). EXs are 30–150 nm diameter vesicles derived from the inward budding of endosomal membranes, resulting in the progressive accumulation of intraluminal vesicles (known as EXs) within large multivesicular bodies (MVBs). These EXs are released to the milieu by fusion with the plasma membrane (8, 9). Ectosomes, also referred as Microvesicles (MVs) are bigger than EXs (100–1000 nm) and they are produced by the direct budding of the plasma membrane (10). Finally, dying cells also shed membranous vesicles, called apoptotic blebs, with heterogeneous shape and size (8). Hence, the different subcellular origin of EVs accounts for their specific composition and function. In this sense, EVs contain a specific subset of common proteins related to biogenesis and trafficking and also a specific signature from their cell or tissue of origin (8, 11), including protein and nucleic acids (4, 12, 13). Therefore, the study of the proteome and the nucleic acid content of EVs may provide information about the cell or tissue of origin and, importantly, their physiological state. Three public online databases including all gathered information about EV content are available: EVpedia, ExoCarta, and Vesiclepedia (2, 14–16).

Urine is a body waste fluid, which can be easily obtained, being therefore an ideal fluid for biomarker determination and analysis. But urine is a complex mixture of filtered and secreted proteins, salts, urea, and metabolites that may vary, not only in physiological situations but specially in diseases associated with renal involvement (17, 18). In addition, the protein composition of the urine is highly dependent on the glomerular filtration rate (GFR), tubular metabolism, tubular reabsorption, diet, and hydration status of the patient among others. The variation in the concentration of a certain protein could be the result of any tissue disorder or pathophysiological alteration. It is estimated that only around 3% of urine proteins are contained in urinary EVs. Therefore, potential interesting biomarkers contained in EVs may be just undetected due to their dilution in whole urine. Thus, the determination of biomarkers specifically related to urinary EVs may be of interest to uncover alterations on the renal system (18).

## Isolation Techniques and Methods to Obtain Urinary Extracellular Vesicles

The complexity of body fluids, and in this case urine, hampers the study of the specific molecular content of EVs.

A previous step to EVs isolation is the management of the sample. Based on the studies of Zhou et al. (19), which focused in the protein content of EVs, it is recommended the use of protease inhibitors in the collection containers to preserve the sample. In addition, an extensive vortex is required to recover the highest amount vesicles, as EVs could remain attached to the plastic. Samples can be either processed immediately or storage at −80°C. Due to the lack of consensus in the published data, the international society of EVs has recently published a position paper, in which they recommend further studies on this issue (6). In addition, as a normal procedure in urine samples, bacterial contamination of the physiologic micro-environment should be avoided (6, 20).

Many studies conducted to date have enriched urinary EVs using methods that do not exclude the presence of non-EV contaminants. However, a gold-standard technique for isolating EVs in the clinical practice is still missing. In this section, we summarize and discuss the different methods used to isolate urinary EVs.

When initiating the isolation of urinary EVs, a first consideration has to be paid to the presence of the Tamm–Horsfall protein (THP)-also known as uromodulin-, a common component of urine samples in both physiological and pathological conditions. THP, among others proteins (such as albumin) that are increased in urine under several kidney pathologies, do interfere with urinary EVs-related biomarkers research and discovery. Therefore, pre-isolation techniques to reduce or eliminate their presence before any EVs isolation/determination are recommended (6, 21). DTT-treatment has been the most widely solution to avoid EV-entrapment by THP (22). An alternative to DTT is the use of 3-[(3-cholamidopropyl)dimethylammonio]-1-propanesulfonic (CHAPS), a mild detergent also proposed to solubilize THP. The main advantage of CHAPS is the preservation of the protein conformation and the enzymatic activity (23). This may be important in experimental designs involving functional activities of urinary EVs. However, it has been also mentioned that CHAPS treatment is more time consuming than DTT-treatment (24). Thus, the use of each method would be directed by the final use of the sample.

Once these major contaminants are reduced, the sample is prepared for enrichment and isolation of EVs. This could be achieved following different methods.

### Ultracentrifugation

Ultracentrifugation was first used to isolate EVs by Jonhstone and Stahl (25, 26), and since then it has been widely used for EVs isolation regardless the origin of the sample. Thus far, most studies found in the literature carry out a two-step differential ultracentrifugation (UC) to isolate EVs: a first low-speed centrifugation at 10,000–17,000 × *g* to eliminate cellular debris, apoptotic bodies, and larger EVs, and a second step at higher speed – ranging 100,000–200,000 × *g* depending on the study-, to pellet smaller EVs. In some cases, this centrifugation method could be complemented or replace with a size filtration step to remove protein aggregates and cell debris. However, some authors consider that filtration step may fracture larger EVs in smaller ones (6).

Under pathological conditions, when glomeruli filtration is compromised, there is a massive loss of protein in urine. These proteins, together with THP and their aggregates could be co-isolated with urinary EVs. To obtain a more purified sample, the pelleted EVs by the differential UC can be processed and resolved by sucrose density gradients to remove contaminants (27). Other density gradients such as potassium bromide or iodixanol (Optiprep™) have been recently proposed to isolate EVs reducing protein contaminations in plasma (28, 29).

Although UC is the technique of reference for basic research in EVs, major drawbacks undermine their applicability in the clinical setting, including the high operator-dependent variability, the lack of a universal protocol, the expensive equipment required, and the low throughput (30).

### Filtration

Filtration is based on passing the sample through a nanomembrane (in general polyethersulfone or PVDF membranes) in a short low-speed centrifugation. This method avoids UC, and can be used with low-volume samples. Cheruvanky et al. analyzed this method as a new option for isolating urinary EVs. They detected that some proteins classically related to EVs were retained by the nanomembrane, thus making necessary an extra-washing step to recover the total amount of EVs (31). In addition, Rood et al. found a low recovery of EVs markers together with co-isolation of proteins i.e., albumin within the urinary EVs pellet (21). Thus far, the protein adherence to the nanomembrane and the high protein retention are important disadvantages for isolating urinary EVs from proteinuric patients using this method. Other options, such as filtration applying vacuum or ultrafiltration, which use a low protein binding membrane, can be used to purify urinary EVs from low-volume samples (32).

### Immuno-affinity and peptide-based isolation

Extracellular vesicles are characterized by the presence of several surface proteins, such as the tetraspanin family. Taking advantage of this proteins expression, several affinity-based methods have been developed to isolate EVs. These methods use antibodies (attached to magnetic beads or other supports) to specific EV proteins (4, 33). Immuno-isolation of EVs still requires a low-speed centrifugation step or magnetic techniques to concentrate vesicles (6, 24).

Ghosh et al. proposed a method for isolating EVs using a synthetic peptide with specific affinity for heat shock proteins, which are described as EVs markers (16). Similar to antibodies, this technique takes advantage of the membrane proteins present on the surface of EVs. The so-called Vn-96 peptide seems to be able to capture EVs from plasma, urine, and cell culture supernatant (34).

Nevertheless, due the lack of unequivocally specific EVs pan-markers, EVs obtained by these methods would render biased samples depending on the antibody or peptide used (9).

### Aggregating agents

Several commercial precipitation reagents have been introduced in the last few years. These reagents such as ExoQuick-TC™, Total EX isolation reagent from Invitrogen™, Exospin™, and miRCURY™ EX isolation kit from cells, urine and CSF, among others, are based on aggregating agents followed by a low-speed centrifugation. The advantage of these methods is their easy application and faster performance, with a huge applicability in diagnostic laboratories and low operator variability (35, 36). On the other hand, their main drawback is the co-isolation of a complex mixture of EVs together with protein aggregates (37) that may interfere with further EV-related marker determination and analyses. Thus, these methods still require of specific pre-treatments to eliminate larger EVs and aggregates (4, 6, 24, 29).

### Size-exclusion chromatography

In the last years, size-exclusion chromatography (SEC) has been applied to fractionate complex biologic samples, such as urine and plasma, and isolate EVs excluding the main contaminants, such as protein aggregates or lipoproteins usually co-isolated by UC. Rood et al. used UC followed SEC to optimize the purity of the urinary EVs. Importantly, this group demonstrated that non-EV-related contaminants could mask the presence of relevant EV markers (21), thus emphasizing the importance of eliminating as much contaminants as possible.

More recently, Muller et al. used this technique for isolating morphologically intact EXs without protein contamination from human plasma samples (38, 39). In our laboratory, we have obtained similar results using urine samples. Thus, SEC can be applied to highly enrich EVs in an efficient manner. In addition, SEC is a medium throughput technique susceptible to be implemented in the clinical setting.

### Microfluidic-based methods

Microfluidic-based methods have been recently developed to isolate EVs. Exochip™ is a microfluidic-based platform based on a polydimethylsiloxane matrix covered with Abs against CD63 (classical exosomal marker) developed for the immuno-affinity isolation of circulating EVs. This method does not require a separation step for quantification of EVs, as the vesicles are labeled with a fluorescent dye allowing their quantification using a plate reader. In addition, RNA extraction or proteomic studies can be carried out as well (40).

Recently, Santana et al. have developed a microfluidic platform to isolate EVs based on their diameter and the deterministic lateral displacement. First assays carried out have shown an efficient separation among EVs subpopulations without altering their biology and morphology. However, the ability to separate EVs aggregates should be deeply analyzed in further experiments. In addition, this technology should also be tested on complex biological samples in which the density of the sample is highly variable (41).

### Hydrostatic dialysis

Musante et al. have recently presented hydrostatic dialysis to isolate urinary EVs without need of UC and as a possible solution to highly diluted samples. This method is based in a dialysis membrane with molecular weight cut-off of 1000 kDa, removing all possible contaminants from EVs samples (36).

In summary, although different isolation methods for EVs have been developed, a standard consensus – with special relevance in the clinical settings – is still missing. This is of special importance due to the variable results obtained when comparing different techniques (37) or even when using different RNA extraction methods, in which the presence of high amounts of RNA could be indicative of non-EV contaminating RNAs (42).

## Urinary EVs as a Source of Biomarkers for Kidney-Related Diseases

Chronic kidney diseases are a public health issue as they cause important morbidity and mortality and impose high economic burden. Several studies suggested that the chronic kidney diseases (CKD) are an independent predictor of mortality risk in the general population (43). In addition, associations between reduced GFR and the risk of death, cardiovascular events, and hospitalization were observed (44).

The replacement therapy for patients with irreversible chronic kidney failure includes hemodialysis or peritoneal dialysis, being the renal transplantation (RTx) by far the modality of choice for those patients (45). Many factors can affect long time graft survival, and despite remarkable progress has been made in the last years, immunologic rejection and adverse effects of immunosuppressive agents still have significant negative long-term consequences.

Currently, kidney function is indirectly monitored by measuring the GFR, creatinine clearance, serum creatinine, and proteinuria. However, these markers are usually a late sign of kidney damage that indicates – rather than predicts – renal dysfunction. Moreover, the unique gold-standard technique to diagnose kidney diseases is renal biopsy. Needless to say that needle biopsies are an invasive non-reproducible technique, and may be associated with patient morbidity. Thus, there is an additional need to find non-invasive alternatives to kidney biopsies.

**Figure 1:**
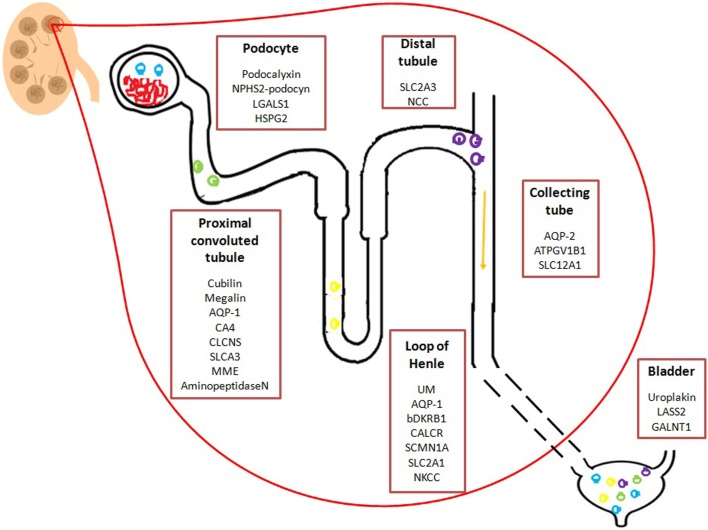
**Schematic representation of potential urinary EVs biomarkers from the urinary tract**. Potential biomarkers identified in urinary EVs from different regions of the nephron, renal tubule, and the bladder. Mentioned molecules are hypothetically related to a specific region of the renal system. *Podocyte:* LGALS1: lectin galactoside-binding soluble 1; HSPG2 heparan sulfate proteoglycan 2. *Distal convoluted tubule*: SLC2A3: solute carrier family 2; NCC: Na-Cl co-transporter; *Proximal convoluted tubule:* AQP-1: Aquaporin-1; CA4: carbonic anhydrase 4; CLCNS: chloride channels; SLCA3: solute carrier family 3; MME: membrane metallo-endopeptidas; *Loop of Henle*: UM: uromodulin; bDKRB1: bradykinin receptor B1; CALCR: calcitonin receptor; SLC2A1: solute carrier family 2; NKCC: Na^+^/K^+^/2Cl^−^co-transporter; *Collecting tube:* AQP2: aquaporin-2; ATP6V1B1: TPase, H transporting, lysosomal 56/58 kD, V1 subunit B; SLC12A1: Sodium potassium chloride co-transporter 2; *Bladder:* LASS2: ceramide synthase 2; GALNT1 polypeptide N-acetylgalactosaminyltransferase 1.

In the case of kidney failure and/or renal diseases, urine is the perfect source of biomarkers for developing new diagnostic tools to identify and stratify patients. Different research programs have been conducted to biomarkers discovery. Initially, efforts were focused to the analyses of total urine, while lately, the study of urine derived EVs have gained interest. Particularly, urinary EVs are secreted from all cell types that face the urinary space (glomerular structures, renal tubule cells, and the cells lining the urinary tract) as represented in Figure 1. It is thus considered that urinary EVs may be a non-invasive image of the physiological state of the renal-tubular system (18, 46). Therefore, an increasing amount of research groups have focused their interest in the study of urinary EVs in kidney diseases. To date, several proteomic studies on EVs have identified proteins that could be associated to kidney diseases (47, 48). Table 1 shows a summary of the current state of biomarkers discovery related to kidney disorders, which are also detailed below.

**Table 1:** **Summary of EVs biomarkers related to kidney disease**.

Pathology	Sample	Isolation method	Biomarker	Reference	Model
**ACUTE INJURE**					
AKI	Spot	DC/200000g	Fetuin A	(76)	Rat and human
	10–16 ml	DC/200000g	ATF3	(49)	Rat and human
I/R	5–8 ml	DC/200000g	Aquaporin-1	(50)	Rat and human
	10–16 ml	DC/200000g	Transcription factor 3	(49)	Rat and human
**GLOMERULAR INJURY**					
FSGS	10–16 ml	DC/200000g	Wilm’s tumor 1	(49, 51)	Rat and human
	45 ml	DC/200000g + SEC	PODXL	(21)	Human
GKD	15 ml	DC	ADAM10	(52)	Cell line and human
DN	450 ml	DC/200000g	miR-130, miR-145, miR-155, and miR-424	(53)	Mice, cell line, and human
	–	Filtra-centrifugation	DPP IV	(54)	Human
	100 ml	DC/175000g	AMBP, MLL3, and VDAC1	(55)	Human
LN	–	DC/200000g	miR-26a, ADAM10	(56)	Mice and human
IgAN	30 ml	SGC	Ceruloplasmin and miR-26a	(57)	Human
TBN	30 ml	SGC	Aminopeptidase A and vasorin	
**FIBROSIS**					
RF	–	DC/2000000	miR-29c and miR-200	(58)	Human
GF	–	DC/200000g	CD2AP, synaptodin mRNA	(59)	Human
**OTHER RENAL DISORDERS**					
CKD	25 ml	Ultrafiltration	mRNA Il-18, NGAL	(60)	Human
Tx	10 ml	DC/200000g	NGAL	(61)	Human
	200 ml	DC/100000g	CD133	(62)	Human
PKD	–	DC/150000g	PKD1, PKD2, PKHD1	(63)	Human
**CANCER**					
MPC	5 ml	DC/100000g	ATGB1	(64)	Cell line and human
			ITGA3		Cell line and human
	–	DC/110000g	miR-34a	(65)	Cell line and human
	200 ml	DC/100000g	PSA, PSMA	(66)	Cell line and human
BC	–	SGC	EDIL-3	(67)	Cell line and human
	–	DC/100000g	LASS2, GALNT1	(68)	Human
	50 ml	DC/100000g	TACSTD2	(69)	Human
RCC	50 ml	DC/200000g	MMP-9, DKP4, EMMPRIN, PODXL	(70)	Human

*Methods: DC, differential centrifugation; SGC, sucrose gradient centrifugation*.

*Disease: AKI, acute kidney injury; I/R, isquemia reperfusion injury; FSGS, focal segmentary glomerulosclerosis; GKD, glomerural kidney disease; DN, diabetic nephropathy; LN, lupus nephritis; IgAN, IgA nephropathy; TBN, thin basement nephropathy; RF, renal fibrosis; GF, glomerulofibrosis; CKD, chronic kidney disease; RTx, renal transplantation; PKD, polycystic kidney disease; MPC, metastatic prostate cancer; BC, bladder cancer; RCC, renal cell cancer*.

*Biomarkers: PODXL, podocalyxin-like; AMBP, α-microglobulin/bikunin precursor; MLL3, histone-lysine *N*-methyltransferase; VDAC1, voltage-dependent anion-selective channel protein 1; CD2AP, CD2-associated protein; NGAL, neutrophile gelatinase-associated lipocalin; ATGB1, GTP-binding protein 1; ITGA3, integrin, alpha 3; PSA, prostate specific antigen; PSMA, prostate specific membrane antigen; EDIL-3, EGF-like repeats and discoidin I-like domain-; LASS2, ceramide synthase 2; GALNT1 polypeptide *N*-acetylgalactosaminyltransferase 1; MMP-9, matrix metalloproteinase 9; DKP4, Dickkopf related protein 4; EMMPRIN, extracellular matrix metalloproteinase inducer; PKD1, polycystin-1; PKD2, polycystin-2; and PKHD, 1 polyductin*.

### Acute kidney injury

Acute kidney injury is a disorder characterized by a rapid decline in the GFR and retention of nitrogenous waste products (71).

Acute kidney injury can be divided in: pre-renal, intrinsic, and post-renal. Pre-renal acute kidney injury (AKI) is the most common cause of AKI and is an appropriate physiologic response to renal hypo-perfusion. The causes of intrinsic acute renal failure (ARF) can be categorized into the following: diseases involving large vessels, diseases of renal microvasculature and glomeruli, ischemic, and nephrotoxic acute tubular necrosis, all processes involving tubulointerstium. Post-renal AKI is due to urinary tract obstruction and accounts for <5% of cases of AKI (72).

Acute kidney injury may be one of the best examples in which serum creatinine concentration (Scr) is used as biomarker of kidney failure. Nonetheless, its concentrations vary widely, and it is not informative in asymptomatic stages and cannot predict the outcome of the disease. It is known that substantial loss of GFR may not manifest with elevations in Scr for several days, and creatinine based estimated GFR is not accurate.

Serum creatinine concentration concentration increases when renal filtration is decreased at least 30%. Moreover, it has been shown that high levels of Scr may not be associated to renal-tubular injury or kidney injury (73). These reasons clearly reflect the need for new more sensitive biomarkers.

Kidney injury molecule-1, neutrophil gelatinase-associated lipocalin (NGAL), or interleukin-18 have been proposed as potential biomarkers for the early diagnosis of AKI in total urine (4, 74). Besides, other groups have recently reported Heat Sock Protein 72 (Hsp72) as putative biomarker, demonstrating an increase in mRNA levels after ischemia and kidney injury (73, 75). Despite these new approaches in biomarkers research, the “classical” parameters already mentioned continue to be used.

As mentioned before, one of the possible causes of acute renal damage is *renal ischemia-reperfusion*. During the ischemia, the absence of nutrients and oxygen creates a hypoxic condition, which promotes the reactive oxidative species formation and inflammatory response once the blood flux is restored. Related to EVs, Zhou et al. identified EVs containing Fenituin-A as potential biomarker after renal injury using an AKI rat model. These EVs were detected not only before any increase in Scr levels but also before any structural change could be detected by kidney biopsy. In addition, this marker was in the same way detected in total urine (76). A few years later, the same group identified transcription factor 3 (ATF3) as an additional EVs biomarker for AKI. Importantly, these biomarkers were only detected on EVs coming from patients and were not present in healthy volunteers (49).

Also, in a rat model for ischemia-reperfusion, Sonoda et al. reported the decrease of exosomal aquaporin-1. Similar results were confirmed in a transplant group of patients (50). The most interesting feature of this biomarker is that they could not find this decrease in EVs in a rat model of nephrotic syndrome, suggesting that this marker could be specific for this pathological state.

### Glomerular diseases

Podocytes are specialized epithelial cells forming the glomerular filtration barrier together with the glomerular basement membrane and endothelial cells. The damage of this structure leads to a loss of proteins and blood cells (77). In glomerular diseases, podocytes are the main cells affected being therefore considered, which podocyte-derived EVs may be a promising source of biomarkers.

#### Diabetic nephropathy

One of the main causes of glomerular disorders is diabetic nephropathy. Related to it, Barutta et al. described a differential expression of 22 exosomal miRNAs between normo and micro-albuminuric patients. Interestingly, the levels of miR-145 and miR-130a in urinary EVs were increased in diabetic and micro-albuminuric patients compared to normo-albuminuric controls. In contrast, miR-155 and miR-424 levels were lower in micro-albuminuric patients whilst normo-albuminuric and healthy controls showed similar levels (53). Then, in mesangial cells cultures, they demonstrated that hyperglycemic conditions induce miR-145 expression. Thus, this miRNA profile represents a novel approach in the search of EVs biomarkers, although further studies with larger cohort of patients are required.

Another interesting putative biomarker for diabetic nephropathy is dipeptidyl-peptidase. This enzyme, which plays a role in T-cell activation, is known to be over-expressed in the plasma of diabetic patients. Its high expression in the kidney took Sun et al. to analyze the vesicular content in a subset of patients and found over-expression of this protein in urinary EVs compared to controls (54).

The quantitative analysis of urinary EVs proteome from diabetic patients carried out by Zubiri et al. showed interesting differences compared to controls. Interestingly, 65% of the proteins detected were identified only in one of the experimental groups. Among those, α-microglobulin/bikunin precursor (AMBP), histone-lysine N-methyltransferase (MLL3) were increased in patients, whilst voltage-dependent anion-selective channel protein 1 (VDAC1) were decreased (55). Hence, these proteins are suggested as promising biomarkers, although more studies are needed for verification.

#### Glomerulonephritis

IgA nephropathy is the most common glomerular lesion of glomerulonephritis whose clinical presentation may vary from hematuria to rapidly progressive glomerulonephritis. Specifically, IgA nephropathy (IgAN) is induced by: aberrant glycosylation of IgA1, synthesis of antibodies directed against galactose-deficient IgA1, binding of the galactose-deficient IgA1 by the anti-glycan/glycopeptide antibodies to form immune complexes and accumulation of these complexes in the glomerular mesangium. Moreover, each step of this pathological process has susceptibility locis proposed by genome-wide association studies but DNA-based test have not been developed (78).

Purification of urinary EVs from IgA nephropathy patients was carried out by Moon et al. (57). These authors identified ceruloplasmin (CP) as biomarker in this disease. In this way, CP concentration appeared higher in IgA patients than in controls; though deepest studies must be perform to confirm these results.

Another important glomerulopathy characterized by podocyte injury is the focal segmental glomerulosclerosis. This pathology could be autoimmune or secondary to obesity and drugs among others. Initial studies by Zhou et al. (49) demonstrated the over-expression of the Wilm’s Tumor 1 protein, a transcription factor required for normal kidney development, in urinary EVs from a mice model with podocyte injury and in humans with focal segmental glomerulosclerosis (49). Some years later, the results were re-evaluated and confirmed by the same group in a larger cohort of patients (51).

Lupus nephritis is a frequent and potentially serious complication among patient with systemic lupus erythematosus whose clinical manifestations are varied being the most common manifestations proteinuria (commonly leading to nephrotic syndrome), microscopic hematuria, and reduced GFR. The histopathology of lupus nephritis (LN) is pleomorphic; based on light microscopic (LM), immunofluorescence (IF), and electron microscopic (EM) findings it can be classified into six classes (79).

Some differences in the levels of several miRNAs have been reported between controls and LN and IgAN patients. Among those, miR-26a is normally found in the glomeruli of control samples, but their levels are decreased in glomeruli from patients. Interestingly, Ichii et al. (56) have reported a higher level of miR-26a in EVs from patients compared to controls, thus suggesting a mechanism for the reduced levels found in patient’s glomeruli. The authors suggested this microRNA could be considered a direct and predictive biomarker of podocyte and glomerular injury.

Similarly, Gutwein et al. studied ADAM10 and its substrate L1, are expressed in differentiated podocytes. Moreover, ADAM10 could be found in urinary EVs and in total urine of LN and IgAN patients but it is absent on healthy donor urine or EVs (52). Although it is not clear the role of this molecule in the kidney or in the EVs, their differential expression suggests they could be considered as possible biomarkers of glomerular damage.

#### Thin basement membrane nephropathy

Thin basement membrane nephropathy is characterized by non-progressive hematuria, minimal proteinuria, and normal renal function due to a thinned glomerular basement membrane. This pathology is thought to be caused by mutation in collagen genes such as COL4 and COL5 (80).

Renal biopsy is the only technique to diagnose between IgAN and thin basement membrane nephropathy (TBMN). In urinary EXs from TBMN patients’, aminopeptidase N, and vasorine precursors were increased compared with IgAN patients. Thus, these precursors could be used as biomarkers in hematuric patients to differentiate between TBMN and early IgAN (57).

### Kidney fibrosis

As mentioned before, CKD is a major death cause worldwide, associated with fibrosis leading to organ failure in the final stages, independently of the primary cause. Lv et al. (58) found a reduced level of urinary EVs miR-29 and miR-200 in a selected group of CKD patients compared to controls. Furthermore, they found that miR-9a and miR-29c could discriminate between mild and moderate-severe fibrosis. Similarly, the same group evaluated other molecules such as CD2AP, NPHS2, and synaptodin mRNA in urinary EVs. They reported an increase of synaptodin and a decrease in CD2AP mRNA levels in patients. As before, their results suggested that CD2AP mRNA levels could reflect tubule-interstitial fibrosis and the glomerulosclerosis degree (59). These results clearly support the idea of using RNA content from urinary EVs as non-invasive tools in the study of renal function and diseases progression.

### Polycystic kidney disease

Polycystic kidney disease (PKD) is a genetic disorder characterized by kidney cystic dilatations that may course with multiple organ involvement. This disease is caused by a dysregulation of PKD1, PKD2, or PKHD1 genes (81, 82). Proteomic studies in urinary EVs by several groups revealed that polycystin-1, polycystin-2, and polyductin (products of the mentioned genes) are easily detectable in patients’ samples, and therefore could be considered for the analysis of the disease (18, 63).

### Renal transplantation

Renal transplantation is the best option for end stage renal disease patients.

The use of urinary EVs as a source of biomarkers for kidney injury after RTx was probed by Alvarez et al. (61). In this study, the authors demonstrated the presence of NGAL in cellular fraction and in urinary EVs from patients. Urinary EVs NGAL detection differed between patients and controls. Interestingly, different quantities of NGAL were detected between deceased and living donors. Thus, suggesting that NGAL could be a biomarker of damage and delayed graft function (61).

Likewise, it has been recently published that other proteins classically associated to kidney injury in total urine could not be considered as EV biomarkers such as kidney injury molecule-1 (KIM-1) and cystatin. These molecules did not shown significant changes in urinary EVs while NGAL mRNA was decreased after transplantation but arose to normal levels in a few days (60); in addition, EV NGAL did not correlate with creatinine reduction. Moreover, in this case, urinary levels of IL-18 and NGAL correlate better with creatinine reduction than EVs markers (60). NGAL, KIM-1, and IL-18 have been proposed as AKI biomarkers as well, but it seems that in the case of RTx, these EVs markers do not correlate with outcome or creatinine level. Thus, the patients’ selection, the degree of the disease and the biomarkers approach play a critical role in the wide of results found between studies. One of the main goals in the search of urinary EVs biomarkers is the reproducibility of the results between different methods and different sample volumes, among others.

The presence of urinary EVs expressing the progenitor marker CD133 has been lately reported by Dimuccio et al. (62). In healthy donors, they could identify two EVs subpopulations based on CD133 expression with a different profile for classical and urinary specific markers. These CD133^+^ EVs were detectable at high levels in urine from healthy donors but not in patients with end stage renal disease. In the case of transplanted patients, CD133^+^ EVs levels were lower than in healthy donors but higher than in chronic patients. The authors considered CD133^+^ EVs as possible biomarkers for tubular function and renal tissue regeneration after transplantation in order to detect possible graft rejection, kidney damage, or incomplete tissue regeneration.

### Renal cell carcinoma

Recently, the comparison of the protein profile of urinary EVs derived from renal cell carcinoma (RCC) patients with control subjects was carried out by Raimondo et al. (70). This study has shown a different protein profile between them. Specifically, in the case of matrix metalloproteinase 9 (MMP-9), Dickkopf related protein 4 and Extracellular Matrix Metalloproteinase Inducer (EMMPRIN), all of them involved in matrix remodeling, and found over-expressed in RCC patients. Moreover, these proteins correlated with the disease progression and the metastatic potential (70).

### Prostate cancer

Benign prostate hyperplasia is one of the most common diseases in men, and only a low proportion of prostate hyperplasias progress to an aggressive disease. The current diagnostic protocol of this tumor includes the serum determination of the prostate specific antigen (PSA), digital rectal examination, and biopsy. This combination could fail identifying properly between aggressive or non-aggressive cancer, leading to an over-treatment of patients (83). Despite urine would be apparently the best biological fluid to find new biomarkers in this pathology, a major effort has been focused in plasma samples (84). Regarding to urine studies, Mitchell et al. found the presence of prostate markers PSA and PSMA in EVs from patients’ samples compared to EV’s from healthy controls (66). Recently, ITGA3 and ITGB1 proteins have been identified significantly more abundant in urinary EVs from metastatic prostate cancer patients compared to benign prostate hyperplasia patients and patients without metastasis (64).

Corcoran et al. (65) described a panel of miRNAs that could be used as biomarker for metastatic prostatic cancer. This panel was set up from studies performed in cell lines in previous publications, and analyzed in human urine samples. The decreased expression of miR-34a is suggested to be a useful tool to discriminate between prostate cancer and benign hyperplasia. In addition, BCL-2, a well-known pro-apoptotic gene, is described as the target of this miRNA (65).

### Bladder cancer

Together with prostate and RCC, bladder cancer is another important disorder of the urinary tract. Recently, it was described that urinary EVs from bladder cancer patients promote tube formation on endothelial cell lines and cell migration; once again EVs are shown to be a vehicle to promote cancer progression. Chen et al. reported the diagnostic potential of the cancer-related protein TACSTD2 in a short cohort of bladder cancer patients (69). Moreover, EGF-like repeats and discoidin I-like domain-3 (EDIL-3) was found to be over-expressed on bladder cancer cell lines (67). Later, the presence of this protein was confirmed in patients’ EVs (67).

Recently, Perez et al. described a different expression profile for four different EV mRNA encoding for LASS2, GALNT1 ARHGEF39, and FOXO3 (68). Briefly, ARHGE39 and FOXO3 were detected only in controls whilst GALNT1 and LASS2 were detected only in urinary EVs from patients. Further studies are required to assess these proteins as bladder cancer biomarkers.

## EVs as a Therapeutic Approach in Kidney Diseases

Research on EVs is not only focused on their potential role as source of biomarkers but also as new therapeutic tools. Taking into account the properties and functions of EVs, different clinical studies have been developed with the aim to use them in therapy (85).

In the context of AKI, only a few studies have tested different sources of EVs for their therapeutic potential. Cantaluppi et al. tested the effect of EVs from endothelial progenitor cells in a rat model for ischemia and reperfusion injury. The miRNA content of these vesicles seems to have a positive effect in tubular cells, reducing apoptosis and promoting cell proliferation (86, 87). Ischemia-reperfusion is characterized by the over-expression of the adhesion molecule MCP-1. During this process, transcriptional repressor activating transcription factor 3 (ATF3), which has an anti-apoptotic effect and inhibit inflammatory state, is induced. Chen and colleagues probed that injection of exosomal ATF3 into model mice, reduces I/R kidney injury (88). Similarly, the effect of liver stem cells EVs in the regeneration of renal tubule injury has been demonstrated in the last months. Sánchez et al. suggest the role of these vesicles in a paracrine mechanism, inhibiting apoptosis of renal-tubular cells in an AKI murine model (89).

## Conclusion

Kidney-related diseases, among others, might clearly benefit of research focused on urinary EVs. These vesicles may concentrate potential biomarkers – otherwise unnoticed by dilution in whole urine – that may reflect the physiological state of the renal system, and which may be relevant in several pathologies affecting the urinary tract, from the kidney to the bladder. However, some issues need to be solved before urinary EVs could be established as a source of biomarkers in the clinical setting. First, it is important to define whether these potential biomarkers should be determined in whole urine or specifically associated to urinary EVs, as this could be related to each pathologic condition. It is considered that RNA and proteins are better preserved in EVs than in urine, being protected from the milieu. Indeed, EVs have the advantage of being directly derived from cells of the renal system, but the disadvantage of needing additional steps. Moreover, due to the different EV isolation procedures used – perhaps biasing the results – it is not clear whether some of the biomarkers identified so far are actually EV-related. Thus, a standard consensus on this subject would be desirable before blind studies on larger cohort of patients are performed to unequivocally identify urinary EV-related biomarkers for kidney diseases. Although most of the results so far are still preliminary, several of the proposed biomarkers have enlighten the way to a better understanding and diagnose of kidney diseases. Besides, as EVs can target and modify the behavior of specific cells, their potential use in therapeutic protocols will merit future research in kidney-related diseases.

## Conflict of Interest Statement

The authors declare that the research was conducted in the absence of any commercial or financial relationships that could be construed as a potential conflict of interest.
